# Assessment of Drug Sensitivity in Hematopoietic Stem and Progenitor Cells from Acute Myelogenous Leukemia and Myelodysplastic Syndrome Ex Vivo

**DOI:** 10.5966/sctm.2016-0034

**Published:** 2016-11-07

**Authors:** Katherine L.B. Knorr, Laura E. Finn, B. Douglas Smith, Allan D. Hess, James M. Foran, Judith E. Karp, Scott H. Kaufmann

**Affiliations:** ^1^ Department of Molecular Pharmacology and Experimental Therapeutics, Mayo Clinic, Rochester, Minnesota, USA; ^2^ Division of Hematology and Oncology, Mayo Clinic, Jacksonville, Florida, USA; ^3^ Division of Hematological Malignancies, Sidney Kimmel Cancer Center, Johns Hopkins University, Baltimore, Maryland, USA; ^4^ Division of Oncology Research, Mayo Clinic, Rochester, Minnesota, USA

**Keywords:** Hematopoietic stem cells, Myeloid progenitor cells, Myelodysplastic syndrome, Acute myelogenous leukemia, MLN4924

## Abstract

Current understanding suggests that malignant stem and progenitor cells must be reduced or eliminated for prolonged remissions in myeloid neoplasms such as acute myelogenous leukemia (AML) or myelodysplastic syndrome (MDS). Multicolor flow cytometry has been widely used to distinguish stem and myeloid progenitor cells from other populations in normal and malignant bone marrow. In this study, we present a method for assessing drug sensitivity in MDS and AML patient hematopoietic stem and myeloid progenitor cell populations ex vivo using the investigational Nedd8‐activating enzyme inhibitor MLN4924 and standard‐of‐care agent cytarabine as examples. Utilizing a multicolor flow cytometry antibody panel for identification of hematopoietic stem cells, multipotent progenitors, common myeloid progenitors, granulocyte‐monocyte progenitors, and megakaryocyte‐erythroid progenitors present in mononuclear cell fractions isolated from bone marrow aspirates, we compare stem and progenitor cell counts after treatment for 24 hours with drug versus diluent. We demonstrate that MLN4924 exerts a cytotoxic effect on MDS and AML stem and progenitor cell populations, whereas cytarabine has more limited effects. Further application of this method for evaluating drug effects on these populations ex vivo and in vivo may inform rational design and selection of therapies in the clinical setting. Stem Cells Translational Medicine *2017;6:840–850*

**Significance Statement**

Despite the fact that malignant hematopoietic stem and progenitor cells play critical roles in the initiation, maintenance, and progression of acute myelogenous leukemia (AML) and myelodysplastic syndrome (MDS), information about the effects of investigational chemotherapeutic agents on these cells is limited. Using the Nedd8‐activating enzyme inhibitor MLN4924 as an example, the authors describe a method for examining the impact of novel chemotherapeutics on these populations ex vivo, demonstrate that MLN4924 is generally more active against AML stem and progenitor cells than the standard‐of‐care agent cytarabine, and report that MLN4924 is also active against MDS stem and progenitor cells ex vivo.

## Introduction

Multicolor flow cytometry has been previously used to distinguish various stem, progenitor, and mature cell populations within the bone marrow [1, 2]. To our knowledge, this approach has not previously been applied to drug sensitivity testing either ex vivo or in vivo. As a result, information about the impact of either investigational agents or standard‐of‐care drugs on stem and progenitor cell subpopulations is limited.

At the same time, there is substantial evidence that malignant hematopoietic stem and progenitor cells contribute to disease initiation, maintenance, and progression, particularly in acute myelogenous leukemia (AML) and myelodysplastic syndrome (MDS) [1, 2, 3, 4, 5, 6, 7]. These malignant hematopoietic stem and progenitor cell populations, which have characteristic surface antigen profiles, have been shown to produce disease upon engraftment, exhibit abnormal differentiation capacity, and outgrow nonmalignant cells [1, 3, 8, 9, 10]. Based on these observations, it is currently thought that targeting one or more of these malignant hematopoietic stem and progenitor populations might be necessary to achieve durable disease remissions in these disorders [2, 4, 5, 11, 12, 13, 14, 15, 16, 17, 18, 19]. Accordingly, an assay that assesses the impact of potential therapeutic agents on malignant versus normal hematopoietic stem and progenitor populations might help identify treatments that have unique activity in AML and/or MDS.

To address this issue, we have developed an ex vivo approach for assessment of drug sensitivity in hematopoietic stem and myeloid progenitor cell populations from AML and MDS patients. Using multicolor flow cytometry to assess malignant stem and progenitor cell populations, we compared the number of live hematopoietic stem cells (HSCs), multipotent progenitors (MPPs), common myeloid progenitors (CMPs), granulocyte‐monocyte progenitors (GMPs), and megakaryocyte‐erythroid progenitors (MEPs) in bone marrow mononuclear cell fractions cultured with drug versus diluent (control) for 24 hours ex vivo.

In this study, we describe the application of this approach using two different agents. Cytarabine, a nucleoside analog that is incorporated into DNA [20] and induces apoptosis [21], is widely used in AML therapy and is thought to be the single most active agent for the treatment of this group of disorders [22, 23]. Unfortunately, up to one third of AML patients fail to achieve a complete remission with cytarabine‐based therapy, and many of those who do achieve a remission subsequently relapse [22, 23]. The second agent, MLN4924, is a first‐in‐class Nedd8‐activating enzyme (NAE) inhibitor that prevents processing of the protein modifier Nedd8, which is required for activation of the Cullin‐RING ligase (CRL) class of E3 ubiquitin ligases [24]. Without the NEDD8 modification, CRLs are inactive and their substrates accumulate, leading to cytotoxicity in neoplastic cells but largely sparing normal cells [25, 26, 27]. MLN4924 has shown promising clinical activity in myeloid malignancies, even in the relapsed or refractory setting [28].

Under the conditions of the ex vivo assay described in this study, MLN4924 is toxic to leukemic and myelodysplastic hematopoietic stem and progenitor cells, whereas cytarabine in general is not. The same assay shows that MLN4924 has limited toxicity in normal hematopoietic stem and progenitor cells, extending a recent report that assessed toxicity of MLN4924 in normal CD34^+^ cells [29]. Thus, the strategy described here might help inform development of future AML and MDS therapies aimed at targeting malignant stem and progenitor cell populations while sparing normal hematopoietic progenitors.

## Materials and Methods

### Chemicals and Reagents

Reagents were obtained from the following suppliers: MLN4924 (Active Biochem, Maplewood, NJ, http://www.activebiochem.com); enhanced chemiluminescence reagents and 16% paraformaldehyde (PFA; Thermo Fisher Scientific, Waltham, MA, https://corporate.thermofisher.com); cytarabine (AraC), heat‐inactivated fetal bovine serum (FBS), dimethyl sulfoxide (DMSO), propidium iodide (PI), Triton X‐100, and Histopaque 1077 (Sigma‐Aldrich, St. Louis, MO, https://www.sigmaaldrich.com); and the broad spectrum caspase inhibitor N‐(2‐quinolyl)valyl‐aspartyl‐(2,6‐difluorophenoxy)methyl ketone (Q‐VD‐OPh; SM Biochemicals, Anaheim, CA, http://www.smbiochemicals.com). Primary antibodies were purchased as follows: Cullin1, Bcl‐x_L_, Mcl‐1, Bim, and glyceraldehyde‐3 phosphate dehydrogenase (Cell Signaling Technology, Beverly, MA, https://www.cellsignal.com); Bcl‐2 (Dako, Carpenteria, CA, http://www.dako.com); Puma (catalog no. sc‐374223; Santa Cruz Biotechnology, Santa Cruz, CA, https://www.scbt.com); and Noxa (Enzo Life Sciences, Farmingdale, NY, http://www.enzolifesciences.com).

Antibodies and reagents for the flow cytometry panel were obtained as follows: APC‐Annexin V, BV510‐CD7, BV711‐CD33, PE‐Cy7‐CD34, PE‐CD47, FITC‐CD123, Brilliant Violet Staining Buffer, Compensation Particles (Antimouse Igκ; BD Bioscience, Franklin Lakes, NJ, http://www.bdbiosciences.com); BV510‐CD3, ‐CD10, ‐CD14, ‐CD15, ‐C19, ‐CD56, ‐CD64, AlexaFluor700‐CD38, PacBlue‐CD45, PerCp‐Cy5.5‐CD90, and Red Cell Lysis Buffer (BioLegend, San Diego, CA, http://www.biolegend.com/); APC‐eFluor780‐CD45RA (eBioscience, San Diego, CA, http://www.ebioscience.com/); and Aqua Fixable Live/Dead Stain (Thermo Fisher).

### Cells and Cell Culture

After institutional review board approval and patient consent, primary MDS and AML bone marrow aspirates were obtained as material in excess of that required for hematopathological examination during routine clinical care. Mononuclear cells from bone marrow aspirates were isolated using a standard Ficoll gradient procedure [30] and resuspended in RPMI 1640 medium containing 10% FBS (medium A) for subsequent experiments. Deidentified umbilical cord blood units containing normal stem and progenitor cells [31, 32, 33, 34] collected from consenting donors and banked for research purposes were obtained from the Colorado Stem Cell Bank. Mononuclear cells were isolated from cord blood units using a previously published protocol [35] and resuspended in medium A.

### Detection of Apoptosis via PI Staining

Cells were exposed to increasing concentrations of MLN4924 in 0.1% DMSO for 24 hours, pelleted, and stained with PI in 0.1% (w/v) sodium citrate and 0.1% (w/v) Triton X‐100 as previously described [36, 37]. After 30 minutes, samples were subjected to flow cytometry using a FACSCanto II (BD Bioscience) to assess PI fluorescence intensity. Analysis and quantification of the sub‐G_0_/G_1_ population were completed using FlowJo software (TreeStar, Ashland, OR, http://www.flowjo.com).

### Western Blotting

Cells exposed to diluent (0.1% DMSO) or increasing concentrations of MLN4924 for 24 hours were pelleted for protein isolation. Sample preparation, SDS‐PAGE, transfer to nitrocellulose membrane, and subsequent protein detection by immunoblotting were performed as previously described [38]. These experiments were conducted in the presence of Q‐VD‐OPh, a broad spectrum caspase inhibitor [39], to limit cleavage or release of proteins and transcripts of interest (due to apoptosis and secondary to loss of membrane integrity) without affecting apoptotic events upstream of caspase activation.

### Quantification of Hematopoietic Stem and Progenitor Cells

Unsorted mononuclear cell fractions isolated from normal donor cord blood units or MDS and AML bone marrow aspirates were counted and plated at a density of 2 × 10^6^ cells in 3 ml medium A per well in 6‐well plates.

Because the baseline number of stem and progenitor cells varies among cord blood samples and among bone marrow aspirates, each cord blood unit or bone marrow aspirate constitutes an independent experiment. Therefore, for each cord blood unit or bone marrow aspirate, one well was treated with diluent (0.1% DMSO) to serve as the control sample for that experiment. Other wells were treated with various concentrations of MLN4924 (30–1,000 nM), consistent with previously published studies [25, 40]. Experiments with AML cells also included a well treated with 100 nM AraC, a concentration that reflects typical serum levels achieved with standard 7 + 3 chemotherapy regimens [41, 42].

After incubation for 24 hours with diluent or the indicated agent, samples were centrifuged at 150*g* for 5 minutes and washed once with cold PBS. Cells were then stained for 25 minutes at 4°C with Aqua Live/Dead stain and the fluorescent antibodies listed in the Chemicals and Reagents section. During this time, compensation beads were prepared in parallel according to manufacturer’s instructions to establish appropriate flow cytometer settings before sample acquisition.

Labeled cells were washed twice in cold PBS, fixed in 2% PFA, and analyzed on an LSRII within 2 hours of preparation. In each experiment, approximately equal numbers of events were collected for each sample (diluent treated and drug treated), with 0.7–1.0 × 10^6^ events collected in various experiments. All analysis was performed using FlowJo software. Cell survival in the drug treated populations was expressed relative to the corresponding diluent treated population.

## Results

### Method for Assessing Hematopoietic Stem and Progenitor Cell Survival in Ex Vivo Drug Sensitivity Testing

The present study was undertaken to compare the effects of cytarabine and the investigational agent MLN4924 in leukemic stem and progenitor cells ex vivo. Normal cord blood mononuclear cells served two purposes in this study: (a) to ensure that the fluorochromes in the antibody panel could be compensated to establish a reliable gating scheme for quantification of stem and progenitor cells, and (b) to test whether MLN4924 or cytarabine was toxic to any of the normal populations of interest.

**Figure 1:**
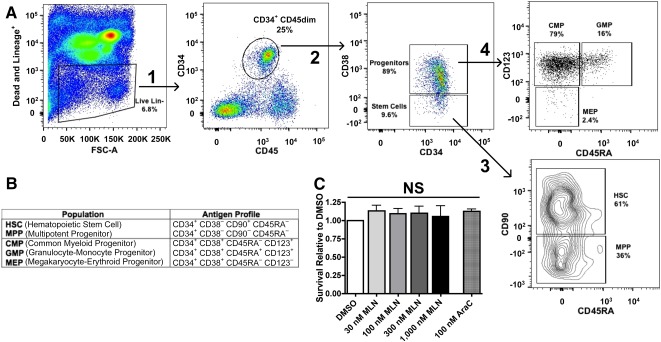
Quantification of hematopoietic stem and progenitor cell populations in cord blood units and relative survival of populations in MLN4924‐treated versus control samples. **(A):** Gating scheme for identification of hematopoietic stem and progenitor cell populations from initial CD34^+^ CD45^dim^ population. See text for details. **(B):** Antigen profiles for each population of interest. **(C):** Survival of CD34^+^ CD45^dim^ cord blood populations in MLN4924 or AraC compared with diluent control. Kruskal‐Wallis test, 6 sample groups, *p* value = NS (.71). Error bars represent ± SEM from four independent cord blood samples. Abbreviations: AraC, cytarabine; CMP, common myeloid progenitor; DMSO, dimethyl sulfoxide; GMP, granulocyte‐monocyte progenitor; HSC, hematopoietic stem cell; MEP, megakaryocyte‐erythroid progenitor; MPP, multipotent progenitor; NS, not significant.

To eliminate dead and differentiated, lineage positive (Lin^+^) cells from analysis, a “dump gate” was established using Aqua Live/Dead stain (emission in the BV510 channel) and BV510 conjugated antibodies against antigens expressed on terminally differentiated cell types. The BV510^−^ population consists of the live and undifferentiated, lineage negative (Lin^−^) cells that will be gated for analysis (Fig. 1A, far left panel). From the Live/Lin^−^ gate, the CD34^+^ CD45^dim^ population containing all stem and progenitor cells was gated (Fig. 1A, arrow 1) and subsequently divided into stem and progenitor populations based on CD38 expression (Fig. 1A, arrow 2). CD34^+^ CD38^−^ cells (stem cells) were divided into HSCs and MPPs based on CD90 expression (Fig. 1A, arrow 3). CD34^+^ CD38^+^ cells (progenitors) were divided into CMPs, GMPs, and MEPs based on CD45RA and CD123 expression (Fig. 1A, arrow 4). The antigen profiles of each population of interest are summarized in Figure 1B. The relative survival of the bulk stem and progenitor cell population (CD34^+^ CD45^dim^) in cord blood samples (*n* = 4) treated with MLN4924 or cytarabine did not differ significantly from the CD34^+^ CD45^dim^ population in the control samples exposed to diluent (Fig. 1C). These results are consistent with a recent report demonstrating lack of MLN4924 toxicity in normal human CD34^+^ cells [29]. In the present study, no decrease in survival was noted in normal HSCs, MPPs, CMPs, or GMPs during MLN4924 treatment (supplemental online Fig. 1). Although some toxicity was noted when normal MEPs were treated with MLN4924, it was less than the toxicity seen with cytarabine (supplemental online Fig. 1).

### MLN4924 Is Cytotoxic to De Novo and Secondary AML Hematopoietic Stem and Progenitor Cells

**Figure 2:**
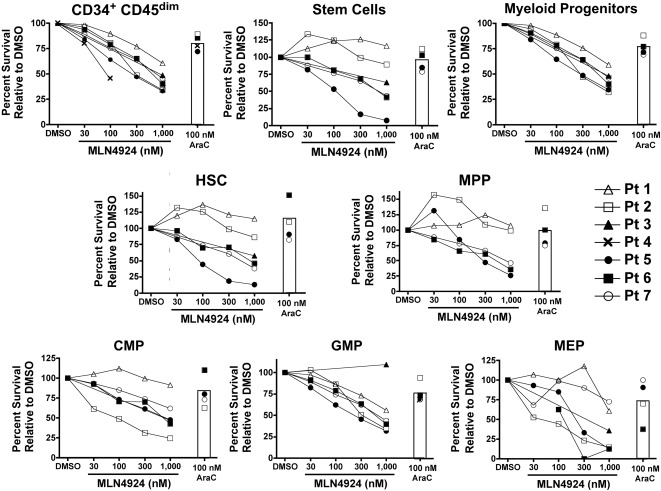
Survival of AML hematopoietic stem and progenitor cells in MLN4924 or cytarabine versus diluent. Aliquots of bone marrow mononuclear cells from AML patients were treated for 24 hours with increasing concentrations of MLN4924, 100 nM AraC, or diluent (0.1% DMSO). Cells were harvested, stained using the fluorescent antibody panel for identification of live stem and progenitor cells (see Methods), and analyzed by flow cytometry. HSC and progenitor populations were quantified using the gating scheme outlined in Figure 1A. Summary of survival of AML stem and progenitor cells relative to populations in diluent treated samples from seven AML patient BMs. Patient data and cell count in each population are reported in Table 1 and supplemental online Table 1 (de novo AML, *n* = 5, patients 1 through 5; post‐MDS AML, *n* = 2, patients 6 and 7). Several populations could not be gated for quantification from patient 4 nor could the MPP from patient 3 (Table 1). CD34^+^ CD45^dim^ = all stem and myeloid progenitor populations; stem cells = HSC + MPP; myeloid progenitors = CMP + GMP + MEP. Abbreviations: AraC, cytarabine; AML, acute myelogenous leukemia; CMP, common myeloid progenitor; DMSO, dimethyl sulfoxide; GMP, granulocyte‐monocyte progenitor; HSC, hematopoietic stem cell; MDS, myelodysplastic syndrome; MEP, megakaryocyte‐erythroid progenitor; MPP, multipotent progenitor.

In contrast to the relative lack of effect of MLN4924 in normal stem and progenitor cells, MLN4924 preferentially diminished populations of leukemic stem and progenitor cells ex vivo. Using samples from seven AML patients (supplemental online Table 1), we compared surviving stem and progenitor cell populations in drug treated samples versus diluent control sample as illustrated in Figure 1. The number of events collected, cell count in the starting Live/Lin^−^ gate used for analysis (i.e., the population used for analysis, as illustrated in the left panel of Fig. 1A), and the cell counts in each stem and progenitor population are reported in Table 1. Results of these analyses are summarized in Figure 2.

**Table 1:** Cell counts in AML stem and progenitor populations

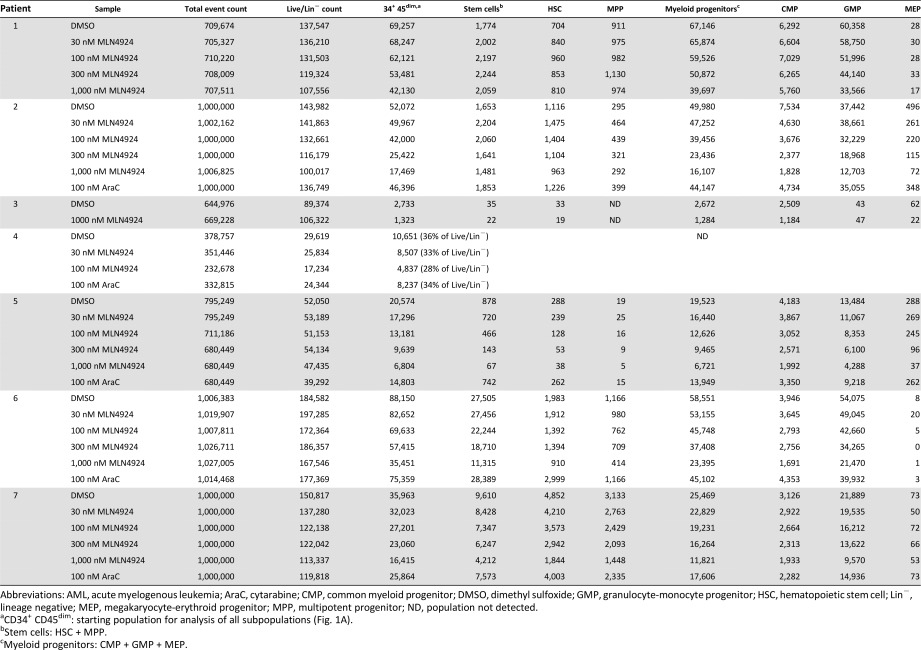

In de novo AML, MLN4924 treatment caused greater reduction of stem and progenitor cell populations than cytarabine did (Fig. 2, patients 1 through 5). However, two instances of resistance to MLN4924 were noted. First, decreased sensitivity to MLN4924 was noted in both stem (HSC and MPP) and progenitor (CMP and MEP) populations isolated from AML patient 1, who had FLT3 ITD^+^ disease (Fig. 2, open triangles; Table 1). Only the GMP population in this AML sample was reduced by MLN4924. Second, in comparison with AraC sensitivity in the same clinical sample, AML patient 2 HSC and MPP populations appeared less sensitive to MLN4924 than the progenitor cell populations (Fig. 2, open squares). Specifically, reduction of HSC and MPP populations below the levels resulting from AraC treatment was not achieved until the MLN4924 concentration in this sample reached 300 nM. However, reduction in progenitor cell populations treated with only 30 nM MLN4924 was equal or greater to the reduction achieved with AraC.

### MLN4924 Is Also Cytotoxic to MDS and Post‐MDS AML Hematopoietic Stem and Progenitor Cells

**Figure 3:**
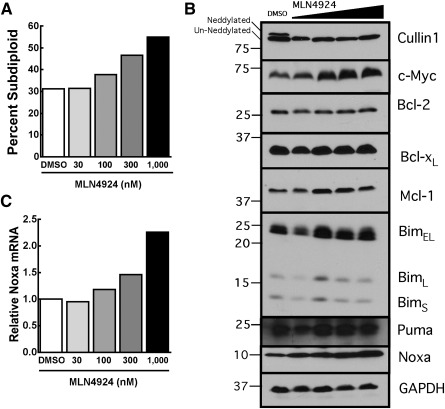
MLN4924 induces apoptosis in post‐MDS AML. **(A–C):** Bone marrow mononuclear cells from a patient with post‐MDS AML were treated with increasing concentrations of MLN4924 versus diluent (0.1% DMSO) for 24 hours. Parallel samples were assayed for subdiploid (apoptotic) cells by propidium iodide staining **(A)**; Cullin1, c‐Myc, and Bcl‐2 family member expression by immunoblotting **(B)**; and Noxa mRNA levels (expressed relative to diluent control) by quantitative RT‐PCR **(C)**. Note the increase in c‐Myc and Noxa as previously seen in de novo AML [27]. Abbreviations: AML, acute myelogenous leukemia; DMSO, dimethyl sulfoxide; GAPDH, glyceraldehyde 3‐phosphate dehydrogenase; MDS, myelodysplastic syndrome.

Our previous biochemical studies showed that MLN4924 treatment of AML cell lines and de novo AML ex vivo results in inhibition of CRL Neddylation, accumulation of the CRL substrate c‐Myc, and c‐Myc mediated transactivation of the *PMAIP* gene that encodes the propapoptotic Bcl‐2 family member Noxa [27, 43]. While performing these studies in de novo AML [27], we observed that MLN4924 also induced apoptosis in post‐MDS AML (Fig. 3A). Similar to de novo AML, post‐MDS AML cells treated with MLN4924 exhibited decreased Neddylation of CRLs (Fig. 3B, top panel), increased levels of c‐Myc (Fig. 3B, second panel), and upregulation of Noxa at the mRNA and protein levels (Fig. 3B, 3C). Although de novo AML and post‐MDS AML are distinct diseases that likely occur through different pathologic mechanisms [44], MLN4924 appears to affect both of these forms of AML similarly.

In light of these results, we applied the ex vivo drug sensitivity assay to marrow mononuclear cell fractions from two post‐MDS AML patients (supplemental online Table 1, patients 6 and 7). As shown in Figure 2, stem and myeloid progenitor cell populations were reduced in a dose dependent manner in response to increasing concentrations of MLN4924 (Table 1; Fig. 2, patients 6 and 7). Furthermore, populations in these two patients were also less responsive to cytarabine.

To follow up these results, we also assessed the effects of MLN4924 on stem and myeloid progenitor cells from patients with various stages of MDS. Given that these populations contribute to disease initiation and progression before transformation to AML, the ability to target them may also be of therapeutic benefit [1, 9, 45, 46]. Accordingly, we examined samples isolated from five patients with MDS of varying risk category (supplemental online Table 2). Again, the number of events collected, cell count in the starting Live/Lin^−^ gate used for analysis (i.e., the population used for analysis, as illustrated in the left panel of Fig. 1A), and the cell count in each stem and progenitor cell population are reported (Table 2).

**Table 2:** Cell counts in MDS stem and progenitor populations

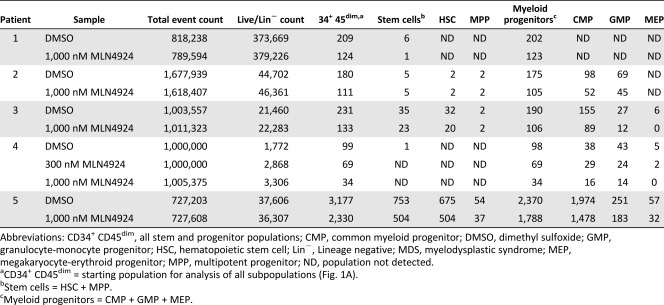

Previous studies conducted to quantify stem and progenitor cell populations in MDS patients showed that the relative distribution of populations correlates with International Prognostic Scoring System MDS risk category [1, 9, 45, 47]. Two observations comparing low/intermediate risk and high‐risk MDS population profiles in our samples were consistent with these reports. First, in the diluent‐treated control samples from low and intermediate‐risk MDS patients 1 through 4, we noted expansion of the myeloid progenitor cell population relative to stem cell population (Table 2; stem/progenitor cell counts: 6/202, 5/175, 35/190, and 1/98 in samples from patients 1 through 4, respectively). In addition, the MEP populations were suppressed in these low‐ and intermediate‐risk cases (Table 2, patients 1 through 4), another observation previously reported in non‐del5q cases in these risk categories [1]. Second, in the sample from high‐risk MDS patient 5, we noted a striking expansion of the stem cell compartment relative to the myeloid progenitor cell compartment (Table 2; stem/progenitor cell count: 753/2370). These observations indicated that relative populations observed in our diluent‐treated samples after 24 hours of culture ex vivo mirrored previous reports regarding alterations in stem and progenitor cell proportions in MDS.

**Figure 4:**
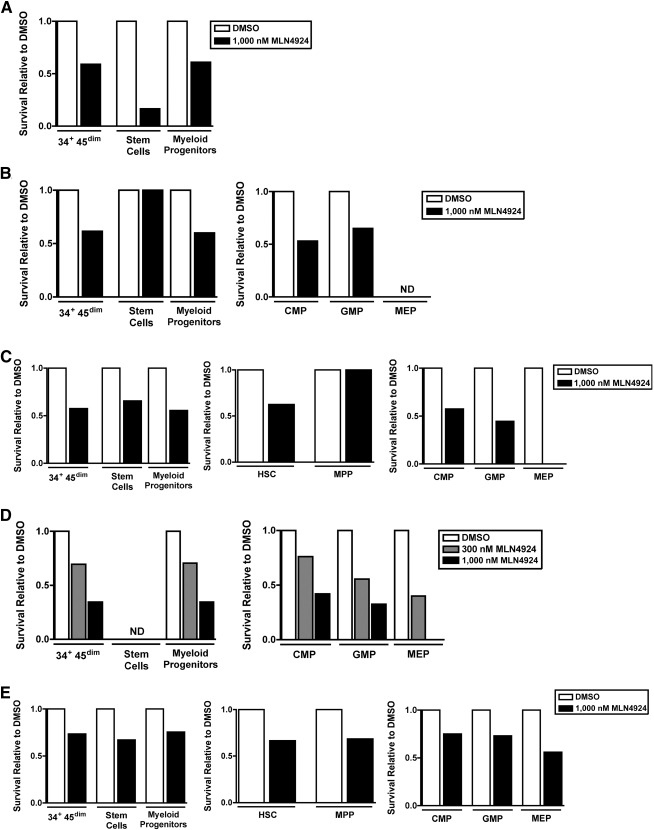
Quantification and relative survival of MDS hematopoietic stem and progenitor cells treated with MLN4924 versus diluent. Bone marrow mononuclear cells were treated for 24 hours with MLN4924 or diluent (0.1% DMSO), harvested, stained using the fluorescent antibody panel for identification of live stem and progenitor cells (see Methods) and analyzed by flow cytometry. HSC and myeloid progenitor populations were quantified using the gating scheme outlined in Figure 1A. **(A–E):** Survival for each population in samples treated with MLN4924 relative to corresponding diluent‐treated population for MDS patients 1 through 5. Patient data and cell count in each population are reported in Table 2 and supplemental online Table 2. CD34^+^ CD45^dim^ = all stem and myeloid progenitor populations; stem cells = HSC + MPP; myeloid progenitors = CMP + GMP + MEP. Abbreviations: CMP, common myeloid progenitor; DMSO, dimethyl sulfoxide; GMP, granulocyte‐monocyte progenitor; HSC, hematopoietic stem cell; MEP, megakaryocyte‐erythroid progenitor; MPP, multipotent progenitor; ND, population not detected.

In Figure 4, survival of cells in the MLN4924 treated samples relative to diluent control sample is reported for measurable populations from each patient. MLN4924 almost universally reduces stem and myeloid progenitor cell numbers in MDS, with one exception being the HSCs from the single low‐risk MDS patient (Fig. 4B). In this sample, the stem cell counts (HSC + MPP) in the diluent control and MLN4924 treated sample were the same (Table 2, patient 2). Admittedly, these are small populations (five cells). However, reduction in other small populations could readily be seen between diluent and MLN4924 treated samples in other patient samples (Table 2; patient 1, stem cell population and patient 3, GMP population). Moreover, dose‐dependent reductions in small populations were noted in the patient 4 samples (Fig. 4D; Table 2).

Although the results are limited by small numbers of stem and progenitor cells in some of the MDS samples, these data collectively demonstrate that drug sensitivity of MDS hematopoietic stem and myeloid progenitor cells can be measured ex vivo. Moreover, although the results indicate some variability in sensitivity to MLN4924 among MDS samples, the majority appear to be sensitive to this agent.

## Discussion

The method we present here for assessing drug sensitivity in MDS and AML stem and progenitor cells contributes a novel dimension to the evaluation of therapeutic agents. Restoring production of functional mature myeloid cells will always be a goal in MDS therapy just as reduction of tumor burden will always be a goal in the treatment of leukemia. However, in the search for agents that produce durable responses, the question is not whether an agent can achieve these immediate goals, but whether it can eliminate the cells responsible for disease initiation, maintenance, and progression. Based on accumulating evidence that stem and progenitor cells play pivotal roles in various stages of MDS and AML pathogenesis [1, 2, 3, 7, 9, 48, 49], it has been suggested that targeting one or more of these populations will likely be necessary to eradicate these disorders [5, 6, 13, 16, 18, 19, 50, 51]. This realization provides the rationale for developing a method to assess the effects of therapeutic agents on MDS and AML stem and progenitor cells.

Several factors influenced the development of the method and studies presented here. First, to diminish cellular stress, decrease processing time, and reduce loss of rare populations that could occur during sorting, the assessment of drug sensitivity in stem and progenitor cells described here (Fig. 1) utilized freshly isolated, unsorted mononuclear cell fractions. Minimizing sample manipulation also reduced the number of variables affecting each sample, allowing changes in stem and progenitor cell number to be attributed to drug exposure. Second, MLN4924 was chosen as a prototype investigational agent based on its continued study in clinical trials for treatment of myeloid disease and our observations of its activity in de novo [27] and post‐MDS AML (Fig. 3).

Contrary to normal isolates treated with MLN4924 (Fig. 1C; supplemental online Fig. 1), stem and progenitor cell populations in de novo and secondary AML were reduced by MLN4924 treatment ex vivo (Fig. 2). Exceptions to this trend occurred in HSC, MPP, CMP, and MEP populations from FLT3^+^ AML patient 1 and the HSC and MPP populations from patient 2 (Fig. 2). Although previous studies in our laboratory and others reported cytotoxic effects of MLN4924 in FLT3 mutated AML cell lines and bulk leukemic cell isolates from patients [27, 52], results from the present study suggest that FLT3^+^ AML stem and progenitor cells may not be as sensitive to MLN4924. Given the need for additional therapeutic options in this patient group, future study of additional samples is warranted. In contrast to this discrepancy between bulk cell population and stem and progenitor cell drug sensitivity assays, the cytotoxic effect of MLN4924 seen in the post‐MDS AML bulk cell isolate (Fig. 3A) was also observed in post‐MDS AML stem and progenitor cells from AML patients 6 and 7 (closed square and open circle, respectively, Fig. 2). Also of note in these two samples, cell counts in HSC, MPP, CMP, GMP, and MEP populations were all reduced in MLN4924 treated aliquots compared with diluent control. Although only two samples were assessed, this result was especially intriguing given that post‐MDS AML patients do not respond optimally to standard cytarabine‐based chemotherapy, with median overall survival of only 6.5 months [53].

Another trend observed in de novo AML stem and progenitor cells is the lack of response to cytarabine (Fig. 2). Although detailed investigation of the mechanisms underlying the observed differences between MLN4924 and cytarabine is beyond the scope of this study, it is clear that the leukemic stem and progenitor cell response to these agents differs. The ex vivo cytarabine exposure utilized in the present study is considerably shorter than the 7‐day treatment often used during induction in vivo and the concentration used is lower than the cytarabine levels transiently achieved during consolidation therapy [54]. Therefore, it is possible that the response of leukemic stem and progenitor cells to cytarabine is greater in vivo. Further investigation is required to assess this possibility.

Cells isolated from low‐ and high‐risk MDS patients (supplemental online Table 2; Table 2) exhibited the relative distributions of stem and progenitor cells previously reported in these two MDS patient groups [1, 9, 45]. Specifically, in diluent‐treated samples from low‐ and intermediate‐risk MDS (patients 1 through 4), greater progenitor cell counts relative to stem cell counts were observed, whereas in high‐risk MDS (patient 5), the stem cell population was expanded relative to the progenitor cell population (supplemental online Table 2; Table 2). These observations provide evidence that the MDS samples examined in the present study are representative of this disorder.

In studies performed on parallel aliquots from these MDS patient samples (supplemental online Table 2), MLN4924 treatment reduced the number of MDS stem (HSCs and MPPs) and progenitor cells (CMPs, GMP, and MEP) compared with their respective numbers present in the diluent control in all five MDS patient cell isolates (Fig. 4). One exception was noted in the stem cell population (HSC + MPP) from patient 2 (Fig. 4B). To our knowledge, this is the first report of MLN4924 activity in MDS samples before leukemic transformation. These results by themselves cannot conclusively establish that only MDS stem and progenitor cells, as opposed to “normal” stem cells, were affected. However, even in low risk MDS patients, cytogenetic and mutation profiling studies have previously demonstrated that the overwhelming majority of HSCs and progenitors are abnormal [45], making it probable that MLN4924 reduction of these populations includes the mutated HSCs and progenitors. Moreover, our study as well as a recently published study from another group [29] indicate that normal stem and progenitor cells are unaffected by the same concentrations of MLN4924 (Fig. 1C; supplemental online Fig. 1).

Although MDS and AML are notoriously heterogeneous diseases, the contribution of malignant stem and progenitor cells to disease initiation and progression is a unifying feature [1, 2, 44, 48, 55]. Defining which specific stem or progenitor cell populations need to be targeted to induce durable remission is an active area of investigation [18]. Furthermore, response or resistance of these malignant stem and progenitor populations has been shown to correlate with clinical course and outcome [1, 2, 6, 8, 56]. Although the strategy presented here was used to study the effects of cytarabine and MLN4924, this method provides an individualized, patient specific approach for ex vivo drug sensitivity testing in leukemic stem and progenitor cell populations that could be applied to other agents. In combination with other tumor profiling platforms and clinical parameters, this approach could potentially be utilized to inform design and selection of therapeutic agents.

## Conclusion

The method presented here enables assessment of drug response in malignant hematopoietic stem and progenitor cells. Because these cells play a critical role in disease initiation, maintenance, and progression, the response of these cells is of special interest when considering the ability of an agent to produce durable clinical responses.

## Author Contributions

K.L.B.K.: conception and design, financial support, collection and/or assembly of data, data analysis and interpretation, manuscript writing, final approval of manuscript; L.E.F.: conception and design, financial support, provision of study material or patients, data analysis and interpretation, final approval of manuscript; B.D.S., A.D.H., and J.M.F.: provision of study material or patients, final approval of manuscript; J.E.K.: provision of study material or patients, data analysis and interpretation, final approval of manuscript; S.H.K.: conception and design, financial support, data analysis and interpretation, manuscript writing, final approval of manuscript.

## Disclosure of Potential Conflicts of Interest

J.M.F. has research support for the C15009 clinical trial (azacitidine & MLN4924, Millennium/Takeda). J.E.K. is a consultant for Tolero Pharmaceuticals. S.H.K. has research funding from Eli Lilly for an unrelated project. The other authors indicated no potential conflicts of interest.

## Supporting information

Supporting Information
