# Supplementary material for: Assessment of Drug Sensitivity in Hematopoietic Stem and Progenitor Cells from Acute Myelogenous Leukemia and Myelodysplastic Syndrome Ex Vivo
Source: Stem Cells Transl Med. 2016 Nov 7;6(3):840–50. doi: 10.5966/sctm.2016-0034 (PMC5442784; doi:10.5966/sctm.2016-0034)
Supplement: Supplementary file 1 — Supporting Information [file SCT3-6-0840-s001.pdf]

Supplemental Material for

Assessment of Drug Sensitivity in Hematopoietic Stem and Progenitor Cells from Acute Myelogenous Leukemia and Myelodysplastic Syndrome *ex Vivo*

Katherine L. B. Knorr, Laura E. Finn, B. Douglas Smith, Allan D. Hess,
James M. Foran, Judith E. Karp, and Scott H. Kaufmann

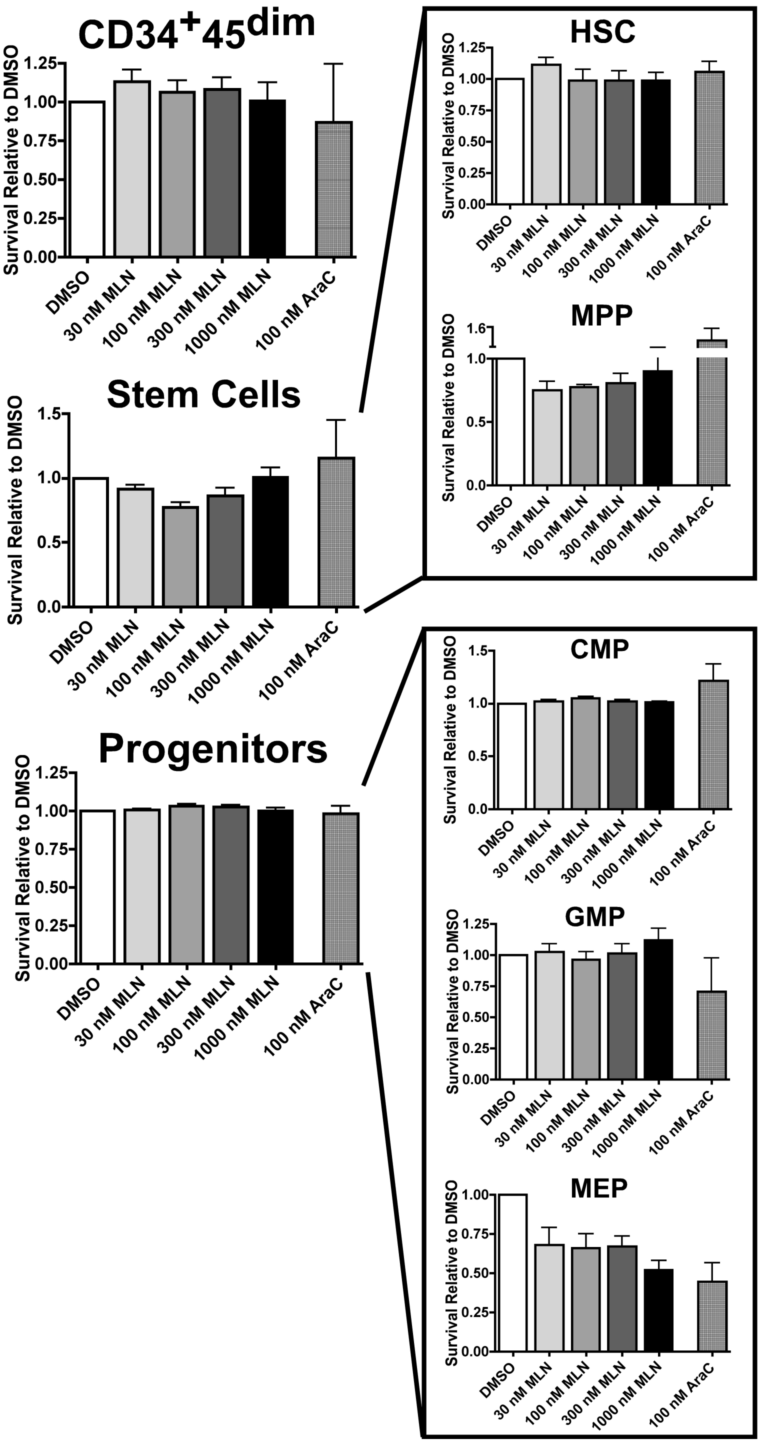

SUPPLEMENTAL Figure 1. Relative survival of hematopoietic stem and progenitor cell populations in cord blood units treated with MLN4924-treated versus cytarabine. After mononuclear cells from 4 different cord blood units were treated with the indicated concentrations of MLN4924 or cytarabine for 24 hours *ex vivo*, survival of the indicated hematopoietic stem and progenitor cell populations was assessed as illustrated in Figure 1. Error bars represent ± SEM from 4 independent cord blood samples.

Supplemental Table 1

AML patient information and disease characteristics

Patient	Age/Sex	Karyotype	*FLT3* Mutation	*NPM1* Mutation	De Novo or Secondary
1	71 M	46 XY<br>t(6;9)	+ ITD	-	De Novo
2	57 M	46 XY<br>t(8;21)	-	-	De Novo
3	53 M	46 XY	-	-	De Novo
4	53 F	46 XY<br>+13 +19	-	ND	De Novo<br>Treatment Refractory
5	74 M	Complex	-	-	De Novo
6	63 M	Complex	-	-	Prior MDS
7	32 F	46 XX<br>t(6:9)	-	ND	Prior MDS

Abbreviations: ITD, Internal Tandem Duplication; ND, not determined.

Supplemental Table 2

MDS patient characteristics and MDS risk category

Patient	Age/Sex	MDS Classification	Prior Treatment	Cytogenetics	Hbg	MCV	WBC	ANC	Platelets	% BM Blasts	Risk IPSS/IPSSR
1	71 M	Unclassified	New Diagnosis	Normal	7.1	109.3	2.1	1.1	22	0.6	Int-1/Int
2	76 M	Refractory Cytopenia Multi-lineage Dysplasia	Procrit	del 20q	8.3	97	3.2	2.6	207	4.4	Low/Low
3	67 M	Refractory Anemia Multi-lineage Dysplasia	Aranesp	Normal	9.9	106.8	2.4	1.5	87	4	Int-1/Int
4	72 M	Refractory Anemia Multi-lineage Dysplasia	Vidaza	Complex	9.7	73	2.2	0.3	84	3	Int-1/High
5	68 M	Refractory Anemia Multi-lineage Dysplasia Ringed Sideroblasts	New Diagnosis	Normal	7.9	103.5	2.2	0.6	10	20	High/Very High

Abbreviations: Hbg, hemoglobin; MCV, mean corpuscular volume; WBC, white blood cell count; ANC, absolute neutrophil count; BM, bone marrow; IPSS, International Prognostic Scoring System; IPSSR, Revised International Prognostic Scoring System.
